# Successful bedaquiline-containing antimycobacterial treatment in post-traumatic skin and soft-tissue infection by *Mycobacterium fortuitum complex*: a case report

**DOI:** 10.1186/s12879-020-05075-7

**Published:** 2020-05-24

**Authors:** Johanna Erber, Simon Weidlich, Tristan Tschaikowsky, Kathrin Rothe, Roland M. Schmid, Jochen Schneider, Christoph D. Spinner

**Affiliations:** 1 Technical University of Munich, School of Medicine, University Hospital Rechts der Isar, Department of Internal Medicine II, Ismaningerstrasse 22, 81675 Munich, Germany; 2 German Center for Infection Research (DZIF), partner site Munich, Munich, Germany; 3 Technical University of Munich, School of Medicine, University Hospital Rechts der Isar, Department of Internal Medicine I, Munich, Germany; 4 Technical University of Munich, School of Medicine, Institute for Medical Microbiology, Immunology and Hygiene, Munich, Germany

**Keywords:** *Mycobacterium fortuitum complex*, Nontuberculous mycobacteria, Chronic wound infection, Bedaquiline, Rapidly growing mycobacteria, Case report

## Abstract

**Background:**

*Mycobacterium fortuitum complex* is a group of rapidly growing nontuberculous mycobacteria (NTM) associated with skin and soft-tissue infections after surgery or trauma. Treatment of NTM is challenging, due to resistance to multiple antimycobacterial agents. Bedaquiline is a diarylquinoline that inhibits mycobacterial ATP-synthase. The drug has recently been approved for the treatment of multidrug-resistant tuberculosis and evidence of its in vitro efficacy against NTM, including *Mycobacterium fortuitum complex*, has been published.

**Case presentation:**

A 20-year-old Caucasian woman with chronic skin and soft tissue infection in the lower leg following a traffic accident in Vietnam underwent a tedious journey of healthcare visits, hospital admissions, empiric antimicrobial treatments, surgical debridement and plastic reconstruction before definite diagnosis of *Mycobacterium fortuitum complex*-infection was established by culture from a tissue biopsy and targeted antimycobacterial therapy was administered. Histopathological examination revealed granulomatous purulent inflammation, which strongly supported the diagnosis. Genotypic identification was performed and broth microdilution for susceptibility testing showed macrolide resistance. Five weeks of induction treatment with intravenous amikacin, imipenem / cilastin, and oral levofloxacin was administered, followed by all-oral treatment with bedaquiline combined with levofloxacin for four months, which was well-tolerated and led to persistent healing with scars but without signs of residual infection.

**Conclusions:**

Bedaquiline is a promising novel agent for NTM treatment, although clinical data are limited and trials evaluating efficacy, safety, and resistance of bedaquiline are required. To our knowledge, this is the first reported case of successful in vivo use of bedaquiline for a skin and soft tissue infection caused by *Mycobacterium fortuitum complex*.

## Background

Nontuberculous mycobacteria (NTM) are mycobacteria other than *Mycobacterium tuberculosis* or *Mycobacterium leprae* that can ubiquitously be found in the environment [1–3]. Depending on the growth rate, NTM can be divided into slowly and rapidly growing mycobacteria (RGM) [1, 4]. The incidence of NTM-related infections is increasing, slowly emerging as a global public health problem [5, 6]. Amongst these RGM, *Mycobacterium fortuitum complex* (MFC) species are predominantly associated with extrapulmonary disease, causing localized skin-, soft tissue-, wound-, or bone-infections following traumatic injuries or surgery [2]. Clinically, the infection may present with subcutaneous nodules and localized recurrent abscesses that often drain spontaneously. Disseminated infections rarely appear and affect predominantly immunocompromised patients [7]. Diagnosis is often delayed as strong suspicion of RGM-infection is inevitable to initiate special culturing from drainage material or ideally a tissue specimen [1]. Accurate genotypic identification of the isolated RGMs to species level is highly recommended, making molecular methods such as DNA hybridization strip assays and gene sequencing essential [1, 8, 9]. As clinical studies of treatment regimens for RGM-infections have not been performed yet [4, 10], no definite standard therapy for MFC-infections has been established to date [4]. Published treatment recommendations and guidelines are mainly based on case studies, clinical experience of experts and antimicrobial susceptibility testing [1, 10]. As with all other NTM-species, MFC-isolates do generally not respond to first-line antituberculous drugs and may be naturally or secondarily resistant to several antimicrobial agents [11–13]. MFC-susceptibility to amikacin, fluoroquinolones, sulfonamides, imipenem, linezolid, cefoxitin, doxycycline, minocycline, and clarithromycin [1] has been reported. However, there is evidence of a high variability in susceptibility to these antimicrobials among different isolates [12]. While macrolides are key drugs for treatment of NTM-related pulmonary disease, in MFC-isolates macrolide resistance via an inducible erythromycin methylase gene (*erm*) was identified despite in vitro susceptibility. Thus, the use of macrolides needs careful evaluation [1, 14]. Due to the intrinsic resistance to various antibiotics, clinical treatment of NTM-related infections is challenging [11, 15, 16]. Especially drug-resistant isolates require potent, well-tolerated and possibly novel antimicrobial agents [11]. Bedaquiline is a diarylquinoline that acts by inhibiting mycobacterial ATP synthase [17, 18]. The agent was FDA-approved for the treatment of multidrug-resistant tuberculosis (MDR-TB) in 2012 [17, 19] and was shown to exhibit activity against several NTM species, including MFC, in vitro [11, 17, 20–22]. Consequently, the use of bedaquiline in combination regimens has been proposed to be a promising therapeutic strategy for NTM-related diseases [11, 15, 23].

## Case presentation

**Fig. 1:**
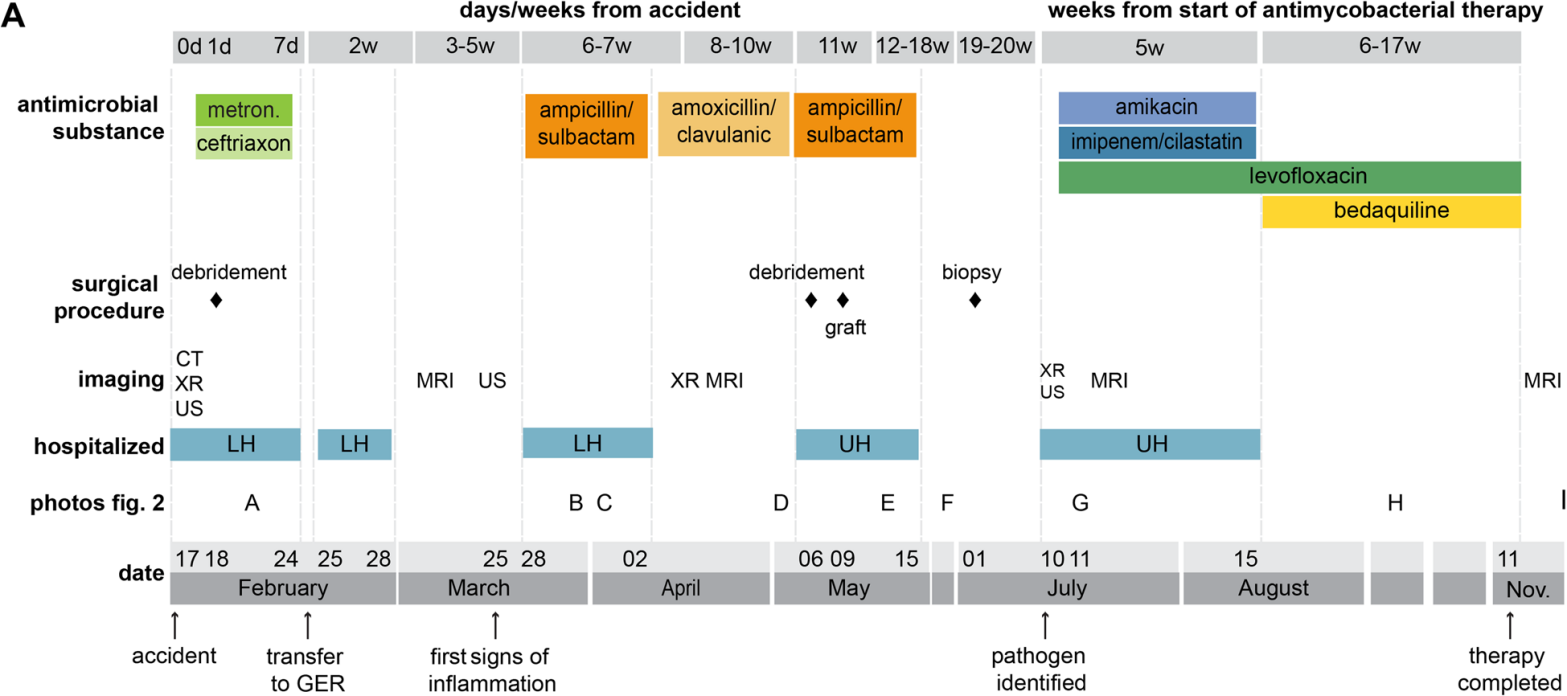
Timecourse of the antimicrobial treatments, surgical procedures, diagnostics and hospital admissions. Links to the respective photos depicted in Fig. 2 have been included. CT, computer tomography; d, day; GER, Germany; LH, local hospital; metron., metronidazole; MRI, magnet resonance imaging; UH, university hospital; US, ultrasound; Nov., November; w, weeks; XR, Xray

**Fig. 2:**
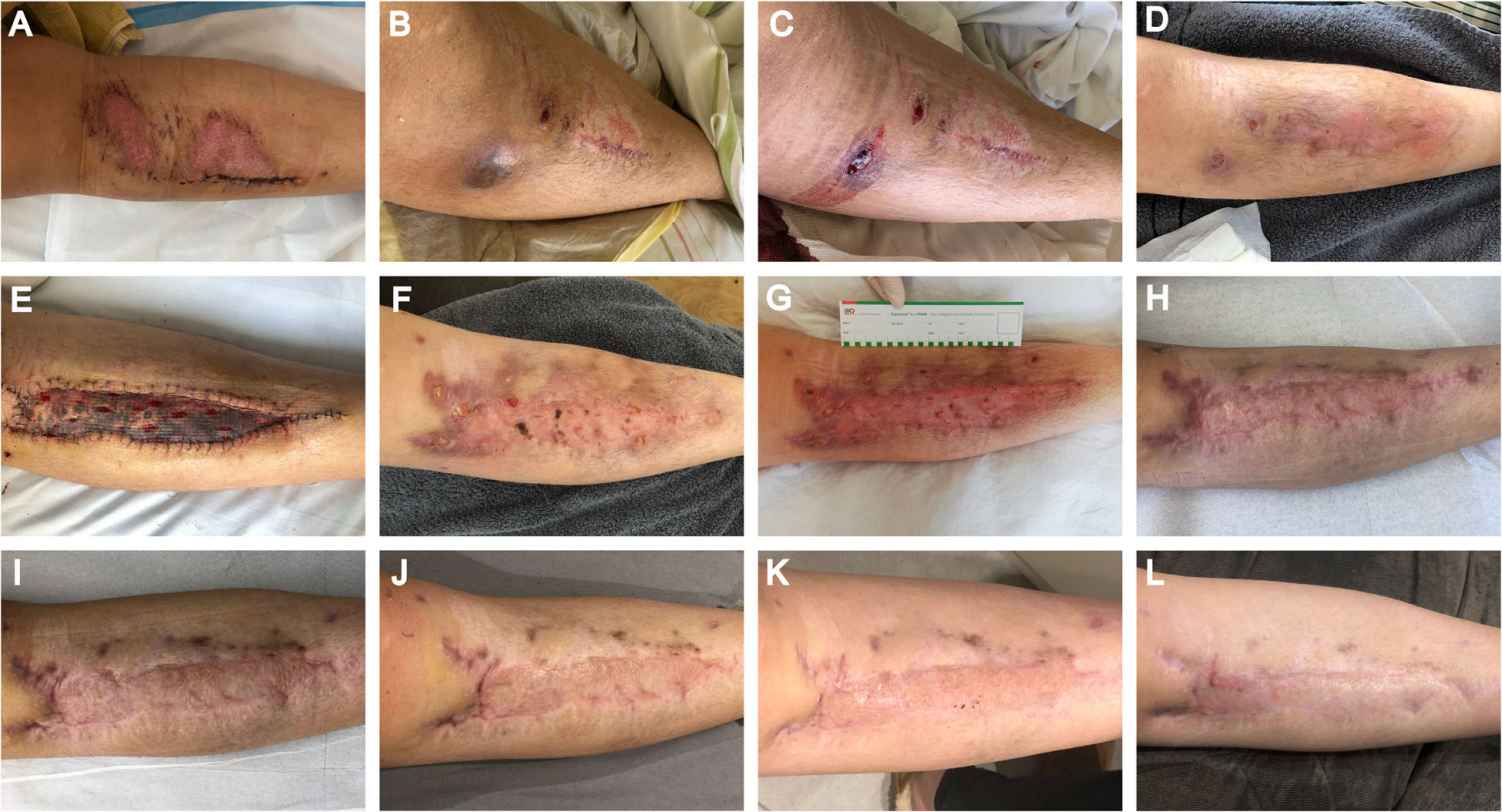
Serial images of the patient’s right foreleg. (**a**) Photograph taken four days after the traffic accident at a hospital in Hanoi following surgical debridement and suture of the wound. (**b**, **c**) Six weeks after the accident a firm subcutaneous nodule was noted (**b**), which drained spontaneously the next day (**c**). The soft tissue infection persisted despite four weeks of empiric antimicrobial treatment (sixth to tenth week after the accident) (**d**) leading to the decision to perform surgical debridement and subsequent skin grafting as well as local flap surgery 11 weeks after the trauma (**e**). (**f)** A tissue biopsy was obtained 19 weeks after the accident when abscesses and nodules reappeared within a few weeks upon surgical debridement and intravenous empiric antibiotic therapy. (**g-i**) After 20 weeks, antimycobacterial treatment was initiated following susceptibility testing of the identified Mycobacterium fortuitum: Photographs show the status before (**g**), eight weeks after antimycobacterial therapy (**h**) and upon completion of the four-month therapy regimen (**i**). The patient was followed-up, continuous improvement was noticed nine months (**j**), 12 months (**k**) and 14 months after the initial accident (**l**)

This case presents a 20-year-old German female patient diagnosed with a chronic wound infection caused by MFC following a traffic accident in Vietnam (see Fig. 1 for a timeline). The patient was travelling through Vietnam when she was involved in a motorbike accident in February 2019. She was taken to the emergency department of a hospital in Hanoi where she presented with a large wound on the right leg. At the time of admission, she was complaining of severe pain in her back and right foreleg, as well as headache and nausea. The patient reported no other pre-existing medical conditions. Radiograph of the lower leg did not show any signs of fracture, and cranio-encephalic traumatic lesions were excluded in the cranial computed tomography (CT) scan. However, a lumbosacral spine CT scan revealed stable fractures of the thoracic vertebrae (T4 / 5). The patient underwent surgical debridement and suture of the leg wound the next day (see Fig. 2a for a representative photograph) and was treated with metronidazole and ceftriaxone over four days. She was discharged eight days after admission, transported to Germany and immediately admitted to a local hospital. At that time, her right lower leg was swollen, but lacked signs of surgical site infection. The patient was discharged three days afterwards, and dressing changes were subsequently performed in an outpatient-setting by her general practitioner and in local hospitals. Approximately five weeks after the accident, wound inflammation was observed for the first time, with redness and warmth around the suture site and dehiscent areas of the wound edges. However, no signs of systemic infection such as fever, increased leukocyte count, or C-reactive protein levels were apparent. Three days later, she was re-admitted to a local hospital with the diagnosis of wound infection. Surgeons described purulent exudate and a subcutaneous firm tumor measuring 2 × 4 cm in size (Fig. 2b) with spontaneous drainage (Fig. 2c). The patient was discharged, and oral antibiotic treatment with amoxicillin / clavulanic acid was continued for two weeks on suspicion of soft tissue infection. In the next follow-up presentation, two new lesions with serous drainage (Fig. 2d) were documented. Of note, several swabs failed to show growth of any pathogenic bacteria on standard microbiological examination. Magnetic resonance imaging (MRI) was performed, showing neither bone involvement, nor a remaining foreign body underlying the chronic infection. The patient was transferred to our university hospital where plastic surgical debridement was performed. On histopathological examination, chronic granulomatous purulent inflammation was documented. The lesion was covered by skin grafting and local flap surgery three days later, while intravenous antibiotic treatment with ampicillin / sulbactam was continued over ten days (Fig. 2e). However, the wound failed to heal, and persistent infected cutaneous lesions were noted on follow-up outpatient appointments (Fig. 2f). MFC could then be grown from a biopsy on Columbia agar on culture day two. Species identification via MALDI-TOF and subsequent genotypic identification using a DNA hybridization strip assay (GenoType Mycobacterium CM, Hain Lifescience / Bruker) [9] was performed and Infectious Diseases specialist consultation was initiated. Minimal inhibitory concentration (MIC)-determination via broth microdilution revealed susceptibility to amikacin and fluoroquinolones, and intermediate sensitivity to imipenem, while the isolated strain exhibited resistance toward linezolid, doxycycline / minocycline, trimethoprim-sulfamethoxazole, clarithromycin, and cefoxitin (based on CLSI-breakpoints, M24-A2 2011). In addition, susceptibility testing for the second line antimycobacterial agents clofazimine, bedaquiline, and delamanid was performed (Table 1). Though official clinical breakpoints for susceptibility testing of NTM for those agents have not been defined yet [4, 15], the results pointed towards resistance against delamanid and susceptibility for bedaquiline considering proposed European Committee on Antimicrobial Susceptibility Testing (EUCAST) breakpoints for *Mycobacterium tuberculosis* (version 10.0) and previous studies [24].

**Table 1:** Drug susceptibility results of the MFC isolate

Substance	MIC	Interpretation
Moxifloxacin	0.25 mg / L	S
Ciprofloxacin	0.5 mg / L	S
Amikacin	4.0 mg / L	S
Linezolid	32.0 mg / L	R
Clarithromycin	16.0 mg / L	R
Imipenem	8.0 mg / L	I
Cefoxitin	128 mg / L	R
Doxycycline	16.0 mg / L	R
Minocyclin	8.0 mg / L	R
Cotrimoxazol	8/152 mg / L	R
Tigecyclin	0.5 mg / L	No breakpoints available
Clofazimine	0.06 mg / L	No breakpoints available
Bedaquiline	0.015 mg / L	No breakpoints available
Delamanid	> 0.5 mg / L	No breakpoints available

The table lists Minimal inhibitory concentrations (MIC) of fourteen distinct antimicrobial substances tested on the clinical MFC isolate. Susceptibility was interpreted using CLSI breakpoints (M24-A2 2011), if available. S = sensitive; R = resistant; I = intermediate.

The patient was transferred to the Infectious Diseases department for further diagnostics and treatment. Due to persistent back pain and mild paresthesia in the peripheral extremities, a whole spine MRI was performed, which showed no sign of disseminated infection. In addition, chest radiograph and abdominal ultrasound did not reveal any abnormal findings and the patient tested negative for Human Immunodeficiency Virus (HIV)-infection. Based on the American Thoracic Society / Infectious Diseases Society of America (ATS / IDSA) guideline and susceptibility testing results, antibiotic therapy was initiated with amikacin (initially 10–15 mg / kg of body weight once a day, intravenously, subsequently adjusted to serum level), levofloxacin (500 mg once a day, orally), and imipenem / cilastatin (0.5 g / 0.5 g twice a day, intravenously) (Fig. 2g). Treatment was generally tolerated well, however, nausea occasionally required symptomatic antiemetic therapy. Also, the patient reported to feel dizzy when exposed to sunlight. After five weeks and upon clinical improvement as well as approval of health insurance coverage and written informed consent on off-label use, the treatment was changed to an oral regimen consisting of levofloxacin (500 mg once a day, orally) combined with bedaquiline used at doses that have been recommended for MDR TB [25] (14 days of 400 mg once a day orally, followed by 200 mg three times per week). The patient could be discharged and was followed up as an outpatient in our Infectious Diseases clinic. Continuous clinical improvement was noted (Fig. 2h). Apart from persisting nausea, the patient did not report any notable treatment-related side-effects. Liver enzymes, renal function and blood count were regularly checked and remained unremarkable. Upon completion of four months of treatment the former wound showed persistent scarring without clinical or radiological signs of infection (Fig. 2i). Since then, the appearance of the scar has continuously improved (Fig. 2j, k) and five months after completion of therapy, which is 14 months after the initial traffic accident, there is no evidence of infection (Fig. 2l).

## Discussion and conclusions

NTM-related infections remain difficult to treat as the range of effective agents is limited and resistance may develop despite combination therapy [15, 16]. The international ATS / IDSA-guidelines recommend at least four months of therapy with a minimum of two drugs with in vitro activity for severe soft-tissue infection caused by MFC, if bone involvement or disseminated disease is absent [1]. For treatment induction, a parenteral regimen consisting of at least two active agents should be administered for two to six weeks. The guidelines recommend to combine an aminoglycoside (preferably amikacin in MFC-infection) with two of the following antibiotics: cefoxitin, imipenem, or levofloxacin [26]. In our case, amikacin and levofloxacin had to be combined with imipenem, as imipenem was categorized intermediate in susceptibility testing (MIC 8 mg / L, Table 1). Prolonged use of amikacin should be avoided, as cumulative dose and treatment duration are risk factors for adverse effects, most predominantly ototoxicity [27–29]. Parenteral treatment induction might be switched to oral combination therapy if compatible with the susceptibility patterns [26], which also facilitates the management of the disease in an outpatient setting [1]. Antimycobacterial agents with sufficient oral bioavailability are limited, particularly in macrolide-resistant MFC-isolates [1]. In the presented case, fluoroquinolones were the only licensed oral treatment option the pathogen was sensitive to. This highlights the importance of sensitivity screening for novel agents, as there appears to be an unmet demand for antimicrobial drugs against multidrug-resistant NTM-isolates [10]. For MDR-TB, several novel and repurposed drugs have successfully been tested and show promising therapeutic effects [30]. Among these drugs, bedaquiline, delamanid and clofazimine have shown activity against NTM in vitro [11, 15, 23, 31–33]. Clofazimine and delamanid were found to be effective against slowly growing mycobacteria (SGM) in vitro [31, 32, 34]. However, they seem to be less active against RGM [11, 34, 35]. In contrast, bedaquiline exhibited clear inhibitory effects against various species of SGM and RGM, including MFC in different studies [11, 12, 17, 20, 33, 36]. Bedaquiline has mostly been described as bacteriostatic but bactericidal activity for several MFC-strains has been reported [20, 22, 36]. Data on MIC-distribution of different NTM species are limited and there are no established clinical breakpoints. For MFC, MICs between 0.007 and 0.25 have been described [20, 36, 37].

In vivo data and clinical experience of bedaquiline in treatment regimens for NTM-related diseases are scarce and restricted to distinct species [11]. Though not consistent, significant activity of bedaquiline has been reported in mice and zebrafish models for *Mycobacterium abscessus*, another species of RGM [37–39]. Human data on bedaquiline is scarce and explicitly limited to a few case studies on the use of bedaquiline in pulmonal disease with *Mycobacterium abscessus*, *Mycobacterium avium* complex and *Mycobacterium intracellulare* [23, 39, 40]*.* However, due to the paucity of data on MFC-infection and novel antimycobacterial drugs, we sought to extrapolate some knowledge from closely related species in order to optimize the oral treatment-regimen for the patient.

Although bedaquiline has not been extensively studied in NTM disease, the drug’s adverse effects have been investigated in various clinical trials for MDR-TB and it has been safely used for even over 18 months [41–43]. However, the long plasma half-life of four to five months and known QTc interval prolongation require careful clinical evaluation [41, 44].

Emergence of resistance is a key problem in NTM treatment. Zweijpfenning et al. have recently published a case where an increase in the bedaquiline MIC led to a resistant phenotype (or sub-population) of *Mycobacterium avium complex* resulting in failure of a bedaquiline-based treatment regimen [45]. We therefore believe that the combination with a highly active flourochinolon to efficiently suppress mycobacterial growth and the preceding intravenous induction therapy are key elements of successful treatment. This combination, however, needs careful evaluation and monitoring of drug interactions, such as QTc interval prolongation. Particularly in individual NTM-drug-regimens based on susceptibility testing a complete check of drug-interactions is important. Further, it will be warranted to monitor bedaquiline resistance through multiple MIC testing in future studies, especially in patients with treatment failure or relapse [46]. In light of preclinical and clinical data and considering the rising incidence of NTM-infections and emerging MDR, bedaquiline may be a promising therapeutic agent as part of combination-regimens to treat NTM infections [15, 23]. However, studies on clinical efficacy are required to confirm this option in the management of MFC-infections. Furthermore, additional research will be needed to potentially establish valid breakpoints for susceptibility testing, to understand resistance mechanisms and to design bedaquiline-containing treatment regimens as synergy for combined treatment with clofazimine and bedaquiline against the RGM *Mycobacterium abscessus* has been shown in vitro [22]. Thus, it will be remarkably interesting to test bedaquiline in combination-therapy to identify promising, potentially synergistically acting combination regimens to maximize its effect.

In summary, to our knowledge, we report the first case of safe and successful in vivo use of oral bedaquiline-fluoroquinolone-combination-therapy in a chronic wound infection caused by MFC. Bearing in mind that clinical studies will be required to gain data on efficacy, safety, and outcome, this case report encourages testing of MFC isolates for susceptibility to bedaquiline and, upon careful clinical evaluation, use of the agent in combination regimens as oral treatment option for MFC and other NTM-associated diseases.

## Data Availability

Data sharing is not applicable to this article as no datasets were generated or analyzed during the current study.

## Abbreviations

ATS: American Thoracic Society
CLSI: Clinical Laboratory and Standards Institute
CT: Computed tomography
EUCAST: European committee on antimicrobial susceptibility
FDA: Food and Drug Administration
HIV: Human immunodeficiency virus
IDSA: Infectious Diseases Society of America
MFC: *Mycobacterium fortuitum complex*
MDR-TB: Multidrug-resistance tuberculosis
MIC: Minimal inhibitory concentration
MRI: Magnetic Resonance Imaging
NTM: Nontuberculous mycobacteria
QTc: Corrected QT interval
RGM: Rapidly growing mycobacteria
SGM: Slowly growing mycobacteria
